# Identification of malaria hotspots in southwestern Benin through spatial joint modelling of malaria incidence and vector abundance

**DOI:** 10.1186/s12936-026-05891-3

**Published:** 2026-04-04

**Authors:** Gabriel Michel Monteiro, Rock Yves Aïkpon, Codjo Dandonougbo, Luigi Sedda, Luc Salako Djogbenou

**Affiliations:** 1 African Institute for Mathematical Sciences Research and Innovation Centre, Kigali, Rwanda; 2 Tropical Infectious Diseases Research Centre, University of Abomey-Calavi, Cotonou, Benin; 3 Lancaster Ecology and Epidemiology Group, Lancaster University, Lancaster, UK; 4 National Malaria Control Program, Ministry of Health, Cotonou, Benin; 5 Department of Vector Biology, Liverpool School of Tropical Medicine, Liverpool, UK

**Keywords:** Anopheles vectors, Malaria hotspots, Sub-Saharan Africa, Spatial epidemiology, Geospatial modelling, Joint models

## Abstract

**Background:**

Identifying the spatial heterogeneity in malaria transmission is crucial for designing geographically targeted control interventions, especially in high-burden communities where hotspot identification and delineation can facilitate the decision-making process toward resource allocation to specific areas where they are most needed. This study is the first attempt to identify malaria hotspots by jointly modelling vector abundance and human malaria incidence, alongside key ecological drivers, providing new insights into entomological and epidemiological synergies for public health management.

**Methods:**

We applied a Bayesian Framework for Joint Gaussian Spatial Processes to log-transformed *Anopheles gambiae* s.l. and *Anopheles funestus* counts, and malaria incidence in eight communes of southwest Benin. Entomological data were obtained from mosquito surveillance activities and routine malaria incidence data from the District Health Information System 2. Malaria hotspots were delineated from a joint risk surface derived from interpolated predictive surfaces of malaria incidence and vectors abundance. Co-regionalization analysis explored local spatial correlations between malaria incidence and each mosquito vector suitability.

**Results:**

Joint risk modelling identified contiguous malaria hotspots located mainly on the western shores of Lake Ahémé, and in Atchannou, Sè, Avloh and Grand‑Popo districts. Four ecological factors emerged as consistent and key drivers for all three processes: wind speed, mid-infrared reflectance, leaf area index and land surface temperature. Contrary to common assumptions, *An. funestus* showed stronger spatial correlation with malaria incidence across 119.95 km^2^ compared to 89.90 km^2^ of *An. gambiae* s.l.; and with 67.29 km^2^ showing synergistic effects of both species.

**Conclusion:**

This study reveals high heterogeneity in the spatial association between malaria and its primary vector species, with *An. funestus* playing a potential prominent role than previously recognized. Our framework offers a useful insight of the distinct ecological preferences of each malaria vector species, and highlights the need for species-agnostic, and spatially targeted interventions informed by entomological and epidemiological data until universal vaccines become widely available.

**Supplementary Information:**

The online version contains supplementary material available at 10.1186/s12936-026-05891-3.

## Introduction

Geographic mapping of diseases is an essential tool in public health. By leveraging Geographic Information Systems (GIS) and geospatial modelling techniques, disease maps facilitate the detection of disparities in disease surveillance, mortality, and burden across different geographic areas [1]. Beyond allowing a thorough understanding of disease patterns, maps can inform policy decisions, by helping to investigate diseases aetiology, and prioritize interventions and services management, for instance, by targeting high risk communities [2].

Disease mapping can utilize various approaches including joint disease modelling which has gained a growing interest in recent years [3]. Although joint spatial modelling of diseases can be based on different statistical models (such as hierarchical models, shared component models, and multivariate spatial models), the term refers to the investigation of the geographic patterns and potential shared determinants, including risk factors or inherent system characteristics, of multiple diseases and/or their components [4]. For example, joint disease mapping allowed for the exploration of relationships between malaria and Glucose-6-phosphate dehydrogenase (G6PD) deficiency [5]. Compared to univariate modelling, joint disease mapping offers the benefit of borrowing and sharing information across diseases and their common risk factors or correlation in space and time, leading to more precise and reliable disease risk estimates, especially in areas with scarce data [6]. Aside from malaria, joint disease mapping has been applied to other health outcomes, including cancers [7], cardiovascular diseases [8], and other vector-borne diseases such as dengue and chikungunya [9].

In malaria research, joint disease mapping has most often been employed as a framework that models malaria in combination with other health outcomes, such as anaemia [10–12] and cutaneous leishmaniasis infection [13]. On the other hand, several researchers have also used joint modelling to investigate inter-related determinants of malaria risk [14]. For instance, in the study conducted in Ivory Coast, Kouame and colleagues used a hierarchical multivariate joint spatially-explicit Gaussian generalized linear model to capture and quantify shared spatial effect and independent risk factors effects for malaria incidence and mosquitoes’ densities [6]. The authors, also by demonstrating that joint modelling can improve predictive estimation compared to individual modelling (non-jointly) propose joint modelling as a standard tool for simultaneous mapping of multiple metrics related to malaria risk as recommended for strategic planning in malaria-endemic countries [15].

One less explored application of joint modelling in malaria research is for hotspot identification. A critical component of a targeted control strategy is the accurate delineation of disease hotspots, which are defined as sufficiently large areas where disease transmission intensity is higher than the surrounding regions and that can be geographically delimited [16–18]. Hotspots are an intrinsic part of malaria transmission biology [19], reflecting the natural heterogeneity common to infectious diseases where a small number of individuals, households or locations carry a disproportionate burden. Hotspot identification and delineation can facilitate the decision-making process toward resource allocation to specific areas where they are most needed, improving overall spending in disease control programs [20, 21]. For these reasons, malaria hotspots have been the focus of public health managers in recent decades in order to maximise the potential control gains that can be obtained from targeting them, particularly in areas where the transmission of malaria is heterogeneous [22].

In Benin malaria is still a major public health concern with 5,128,000 cases and approximately 10,149 deaths in 2023 [23]. There is also a growing body of literature highlighting the heterogeneity in malaria transmission in the country [24, 25], which is likely hindering efforts for the control of the disease [26]. Malaria hotspots identification can therefore help in the optimal allocation of public health resources and the development of effective intervention programs in Benin [21].

To the best of our knowledge, in Benin, no one has investigated malaria hotspots or analysed malaria risk from a joint entomological and epidemiological perspective, although some studies employed spatial modelling to map malaria or its vectors [27, 28]. This is despite a growing body of evidence showing increased heterogeneity in the disease transmission, which contributes to hotspot formation [19], and reinforces the need for geographically-targeted interventions in areas most in need of malaria control. This lack of evidence on the areas at highest-risk of malaria limits the ability of health authorities to target resources and tailor interventions where they are most needed.

This study addresses this gap by introducing a joint modelling approach that leverages mosquito vector abundance and malaria incidence data to delineate malaria hotspots in southern Benin. We adopted the Framework for Joint Gaussian Spatial Processes (FJGS), a hierarchical multivariate spatial model that estimates shared spatial structures and shared and independent risk factors, here applied to two major primary vectors and malaria incidence. The role of the shared random effect in this framework is to capture the unknown common geographic factors that jointly affect all processes. Through joint modelling we investigated similarities and differences in environmental effect between malaria incidence and malaria vectors for a highly endemic malaria area in South Benin; with the main aim to identify malaria/mosquito hotspots for target interventions. Our final goal is to support the Benin National Malaria Control Programme and other local public health managers in identifying priority areas for geographically-targeted malaria control strategies, and offer a framework to other malaria-endemic regions and countries to optimise their interventions.

## Materials and methods

### Study area

**Fig. 1:**
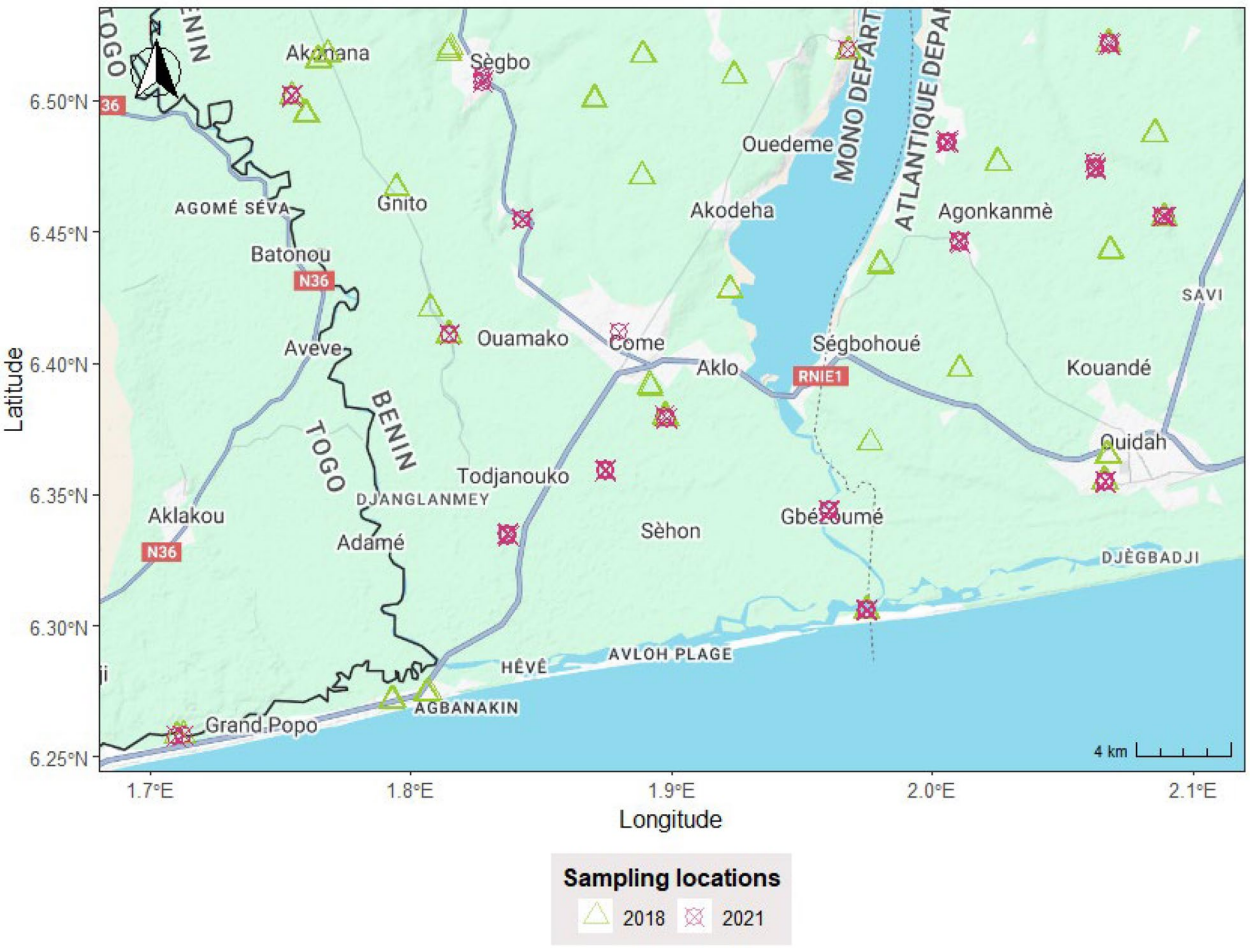
Study area and surveyed locations. Basemap source: Google Maps. Map data ©2025 Google

The study site encompassed eight communes located in Southwest Benin. These communes were Athiémé, Bopa, Comè, Grand Popo, Houeyogbé, which are part of the Mono department; and Kpomassè, Ouidah, and Tori-Bossito, situated in the Atlantique department. This region is a coastal zone with lakes and lagoons (Fig. 1), characterized by a sub-equatorial climate with two rainy seasons (a longer and intense rain period from April to late July, and a shorter and less intense rainy period from mid-September to November); and two dry seasons (mid-July to September and December to April) [29]. The average temperature is 28.9 °C, the average relative humidity is 76%, and the total annual rainfall averages 1300 mm. Malaria transmission in this region is perennial, meaning it occurs throughout the year. The endemicity levels in these areas can be characterized as mesoendemic to hyperendemic, indicating moderate to high transmission rates depending on the season [30, 31]. The main malaria vectors identified in this area include *Anopheles gambiae* s.l. and *Anopheles funestus* [24].

### Entomological data

Mosquitoes’ collection was carried out in two surveillance phases using CDC light traps (Model 512, John W. Hock Company, Gainesville, FL) [32]. Phase 1 of the collection was conducted from late September to November 2018 in 30 villages for a total of 120 houses (four houses per village) surveyed five times. Houses were randomly selected following a spatially-balanced sampling design (lattice with close pairs) [33], which aimed to maximize spatial coverage (lattice) and improve model inference (close pairs) while accounting for different ecological conditions (sampling sites are allocated to ecological zones or strata proportionally to the strata total area). A spatially-balanced sampling design was used for the first phase because of the lack of entomological data for the study area. Mosquitoes’ collection for phase 2 was carried out in 2021 following a similar calendar period of Phase 1 (October–November) and was based on a spatial adaptive sampling design fully described in [27]. This approach targeted areas with the highest risk of detecting *Anopheles gambiae* s.l. and the highest uncertainties, based on Phase 1 data prediction results, to improve the accuracy of the mosquito distribution. The spatial adaptive sampling design allocated 17 villages for mosquitoes’ collection, 9 of which were the same as from phase 1 while 8 were newly sampled villages (Fig. 1) [27]. In each village, 4 houses were surveyed (totalling 68 houses), with mosquito collections conducted over four consecutive nights each week for four weeks. We then summarized nightly counts into a weekly mean per household to harmonize sampling effort across nights.

During the two sampling phases, a volunteer from each village was identified and trained in the use of the CDC traps and sampling protocol. Sampling female mosquitoes that are actively seeking a blood meal was facilitated by positioning the traps in a room approximately 1.5 m from the ground, and adjacent to an occupied bed, covered by a net, from 18:00 to 07:00 the next morning. The order of house collection was randomized before each weekly collection to remove systematic biases. Each morning, mosquitoes were collected from the traps, stored in Ziplock bags containing silica gel and transported immediately to the laboratory of the Tropical Infectious Diseases Research Center of University of Abomey-Calavi in Ouidah (https://www.cremituac.org/) for morphological identification using available keys in the literature.

### Malaria incidence data

Monthly malaria cases data was retrieved from the District Health Information System (DHIS2); and was available at third-level administrative division (arrondissements), for all 34 arrondissements falling within the study area and for the period relative to mosquitoes’ surveillance activities. The population size at the arrondissements was provided by the Statistics and Demography Office of Benin. Malaria incidence at arrondissement level was calculated as follows:

$$malaria\;incidence = \left( {\frac{confirmed\;malaria\;cases}{{population}}} \right) \times 1000$$

### Environmental variables

Environmental predictors were obtained from satellite data repositories at varying spatial resolutions allowing to capture different spatial scale-dependent effects (see supplementary Table S1), or in other words, to account for factors acting at very local geographic scales and others at large scale. Datasets on proximity to running and stagnant water bodies were derived from OpenStreetMap waterway shapefiles (~ 1 km resolution). Soil texture and soil drainage at depth 0–5 cm were sourced from the ISRIC—World Soil Information Portal, with a spatial resolution of 250 m. We also included 10-m resolution global land cover classification extracted through Esri Sentinel-2 Land Cover Explorer (Copernicus), while topographic variables such as elevation and slope at 30 m resolution were derived from the NASA Terra Advanced Spaceborne Thermal Emission and Reflection Radiometer (ASTER) Global Digital Elevation Model (ASTGTM). Key climatic variables, including precipitation, temperature (minimum and maximum), wind speed, soil moisture, and runoff, were derived from the TerraClimate website at approximately 4-km monthly resolution. We also included satellite MODIS datasets such as daily Land Surface Temperature and Emissivity (MOD11A1) at 1 km resolution; 16-day composite vegetation indices EVI and MIR reflectance (MOD13A2) at 1 km resolution; and 8-day composite net evapotranspiration (MOD16A2GF), and 8-day composite Leaf area index (MOD15A2H) both at 500-m resolution. Except for land cover, elevation, and water proximity variables, the amplitude and variance of all predictors obtained at different timeframes were calculated at their native spatial resolutions. The environmental factors described above are all known to influence mosquito habitat suitability and/or malaria transmission dynamics [34].

### Ethical approval and field study permission

The malaria data consisted of aggregated case counts reported through the national health information system and did not include individual-level data; therefore, no ethical approval was required. For entomological collections, household heads were informed about the study’s objectives, and written consent was obtained before mosquito sampling in their homes. Signed consent forms are securely stored at TIDRC/UAC for 10 years starting in 2021. This research did not involve animals or interviews with human participants, and no specific licenses or permits were required. Patients and the public were not involved in the design, conduct, reporting, or dissemination of the research. Findings will be shared with the National Malaria Control Programme (NMCP) of Benin to inform subnational tailoring of malaria control interventions.

### Statistical analysis

In this study, the two *Anopheles* species, *An. gambiae* and *An. funestus,* and malaria incidence were modelled using a log-Gaussian generalized linear model. These three models were jointly parameterized under the assumption of a shared but unknown spatial process, meaning they all relied on the same function for the spatial autocorrelation [6]. For each model, explanatory variables were identified through a separate selection process (detailed below). Once important variables were identified, we proceeded with joint modelling of the vectors and malaria incidence data, followed by model evaluation, validation, and a co-regionalization analysis of predicted values and associated uncertainty. Co-regionalization occurs when the joint suitability of both vector species, or a vector and malaria incidence, overlap within a given region—this is reflected in the cross-modelled correlation in suitability values [6].

Finally, we conducted a malaria hotspot analysis to identify areas where malaria risk was consistently high relative to the contributing mosquito vector species, integrating insights from co-regionalized patterns of vector suitability and malaria incidence. This allowed for a more comprehensive understanding of spatial risk factors and potential areas targets for interventions.

All statistical analyses were conducted in R statistical software (version 4.4.1), using the following packages: MuMIn for model selection, bmstdr and spbayes for model inference and prediction, and coda for convergence assessment.

#### Variable selection

Collinearity is a common issue among ecological variables and can lead to poor model-based variable selection due to redundancy. To address this, we first screened for collinear predictors prior to variable selection by excluding those that had an absolute Pearson’s correlation coefficient above a value of 0.7 with any other predictor. | r |> 0.7 has been demonstrated to be an appropriate indicator for when collinearity begins to severely distort model estimation and subsequent prediction [35]. Six out of the 42 predictors were found collinear with other variables and then removed, retaining those that in turn had higher significance in predictor coefficients, greater explained variance, or lower AIC values in univariate general linear models. For each model, we performed a forward stepwise variable selection [36] using a spatial Gaussian generalized linear model which tested 34 predictors: 33 environmental factors, one health facility attendance rate variable, and additional two *Anopheles* species abundances for the malaria model (see supplementary information S5). Beginning with a baseline model, predictors were evaluated one at a time for inclusion, and the predictor yielding the lowest value of Watanabe-Akaike Information Criterion (WAIC) was added at each iteration. This process was repeated until further additions did not improve WAIC. The selected model was the one with the lowest WAIC among the models explored during this sequential process [37]. WAIC accounts for the full posterior distribution rather than relying on a single point estimate [38]. The same model type was used during the variable selection process as in the joint modelling framework in order to ensure consistent results [39].

#### Joint modelling

In this study, we employed the Framework for Joint Gaussian Spatial Processes (FJGS), a Bayesian hierarchical multivariate joint spatially explicit log-Gaussian generalized linear model [6]. Within this framework the log-transformed counts of *An. gambiae* and *An. funestus*, alongside malaria incidence, were modelled as three interdependent spatial processes sharing a common (joint) spatial structure. In other words, *Anopheles* mosquitoes’ abundance and malaria incidence are assumed to jointly depend on the distance between sampled locations, i.e. that sites closer to each other tend to exhibit more similar mosquito populations and malaria incidence rates compared to those farther apart. The full mathematical description of the model can be found in [6], and in the supplementary information S5. Convergence was evaluated using Gelman and Rubin's potential scale reduction factor and the Geweke time-series statistics [40]. 95% credible intervals were calculated for each parameter estimate using equal-tailed intervals.

#### Model validation

We assessed the model’s predictive performance using three complementary metrics: root mean square error (RMSE), mean error (ME), and coverage. RMSE evaluates the accuracy of point predictions, ME assesses systematic prediction bias by measuring the average difference between predicted and observed values, and coverage assesses how well the 95% prediction intervals captured the true values [41, 42]. To assess predictive performance under spatial dependence, we performed spatial cross-validation by selecting approximately 10% of the data per week, ensuring each week contributed at least one validation sample. The validation samples were chosen to be spatially representative, covering a range of pairwise distances. These held-out observations were then used to evaluate both the accuracy of the central predictions and the reliability of the model’s uncertainty estimates, by checking how many actual observations fell within the MCMC-derived 95% prediction intervals. A coverage value of approximately 95% is generally considered ideal, with values exceeding this potentially reflecting overly conservative prediction intervals [43].

#### Suitability, co-regionalization and hotspots identification analyses

Suitability maps were derived from the fitted joint spatial models using posterior predictive inference. For each process, predictions were generated over a regular grid covering the study area at a spatial resolution of 1 km × 1 km using the spPredict function from the spBayes R package, drawing on posterior mean fitted values at the sampling locations. At each grid cell, the posterior mean of these distributions was interpreted as the estimated suitability surface, while the posterior standard deviation was used to characterise predictive uncertainty. For malaria incidence, predictions were conditional on the predicted *An. gambiae* abundance to preserve the structure of the model. The resulting continuous surfaces of suitability and uncertainty were obtained using multilevel B-spline approximation via the mba.surf function from the MBA R package.

The modelling framework does not assume a fixed correlation for the three processes, but instead a common shared effect between them. To estimate the overall correlation between the processes, i.e. accounting for the fixed effects and random effects together, we perform a co-regionalization analysis. Since the suitability predictions were obtained from a joint model, direct correlation estimation was possible. Local correlations between the estimated suitability surfaces for each vector (*An. gambiae* and *An. funestus*) and malaria incidence—and similarly between the prediction’s uncertainties—were assessed by employing a moving window correlation analyses. For each pixel in the interpolated surfaces, a local window of 5 × 5 km was defined, corresponding to a 10 × 10 cell neighbourhood centred on the focal pixel. Within each of these local neighbourhoods, we extracted the estimated values of suitability (or uncertainties) for both malaria incidence and vector species. Pearson’s correlation coefficients were then computed between the two corresponding sets of datasets—either suitability or uncertainties—from the malaria and vector surfaces. These correlation values were mapped across the study area to visualize the spatial patterns of local association. Grid cells were classified into four distinct categories (AG, AF, AG + AF, and Uncertain) based on local associations. Cells labelled AG correspond to locations where malaria uncertainties were locally and positively correlated (correlation coefficient > 0.2) with *An. gambiae* uncertainties, conditional on both residual surfaces exceeding their respective 25th percentile thresholds. AF denotes analogous locations for *An. funestus*. The AG + AF category identifies areas where malaria uncertainties were simultaneously correlated with uncertainties of both vector species under the same correlation and quantile criteria. Cells labelled Uncertain correspond to locations where the criteria for classification into the AG, AF, or AG + AF categories were not met.

For hotspots delineation, we computed a joint malaria risk defined as the product of predicted malaria incidence and the probability of vector presence:

$$Joint\;malaria\;risk = malaria\;incidence \; \times \;probability\;of\;Anopheles\;presence$$

where malaria incidence is the surface obtained from the posterior predictive mean of malaria incidence. The probability of *Anopheles* presence was computed by applying a probabilistic sum operation (as used in probability theory to compute the probability of union events) to species-specific probabilities for *An. gambiae* and *An. funestus*. The probability surface of each species presence was obtained by evaluating the Gaussian cumulative distribution function at the posterior predictive mean, using the empirical mean and standard deviation of the observed data at sampled locations. This yields the probability that predicted abundance exceeds the typical observed level. The resulting joint malaria risk surface was normalized to a 0–1 scale, and classified into four categories using empirical thresholds determined from the distribution of the joint risk values: “Very high intensity” (≥ 0.8), “High intensity” (< 0.8– ≥ 0.6), “Medium intensity” (< 0.6– ≥ 0.3), and “Low intensity” (< 0.3), to facilitate spatial delineation of malaria transmission hotspots and guide targeted intervention strategies. This empirical thresholding approach is consistent with previous malaria risk mapping studies, such as in Mozambique, where hotspots were classified as areas with ≥ 80% probability of exceeding a specified prevalence threshold [44].

## Results

### Summary statistics

In both years, *Anopheles gambiae* was markedly more abundant than *Anopheles funestus*, with mean mosquito counts per household per night of 4.8 in 2018 and 1.8 in 2021 for *An. gambiae*; and 0.2 and 0.6 for *An. funestus*. Malaria incidence showed a higher average in 2018 (60.1 per 1,000 persons) than in 2021 (39.1 per 1,000), with maximum values reaching 139.4 and 79.7, respectively. Similarly, the total number of malaria cases was substantially higher in 2018 (508,919) compared to 2021 (141,984). These key characteristics of the study populations, including vector abundance (only female mosquitoes were counted owing to very few males trapped during collections) and malaria burden across study villages in 2018 and 2021, are presented in Table 1. The observed trend of temporal decline in both mosquito abundance and malaria incidence may reflect broader factors such as changes in environmental conditions or a potential impact of malaria control interventions.

**Table 1:** Summary statistics of the study populations

Year	Variable	Min	Median	Mean	Max	SD	Total
2018	*An. gambiae*	00	1	4.804	213	12.458	3454
	*An. funestus*	0	0	0.214	23	1.288	154
	Human malaria (incidence per 1000 persons)	16.671	44.081	60.125	139.437	36.678	–
	Human malaria (counts)	94	612	707.815	2049	510.873	508,919
2021	*An. gambiae*	0	0	1.768	34	4.866	481
	*An. funestus*	0	0	0.563	13	1.559	153
	Human malaria (incidence per 1000 persons)	7.913	36.564	39.116	79.682	18.581	–
	Human malaria (counts)	91	387	522	2115	440.093	141,984

Spatial patterns of vector abundance revealed pronounced heterogeneity across the study area (Fig. S2), with higher vector densities observed in the western part of the study area. In contrast, northeastern villages presented lower and more scattered mosquitoes’ catches, particularly in 2021. Malaria incidence similarly exhibits a heterogeneous spatial pattern, reinforcing assumptions of geographic variation in malaria transmission risk in addition to the temporal variation described above.

### Suitability, co-regionalization and hotspots prioritization maps

**Fig. 2:**
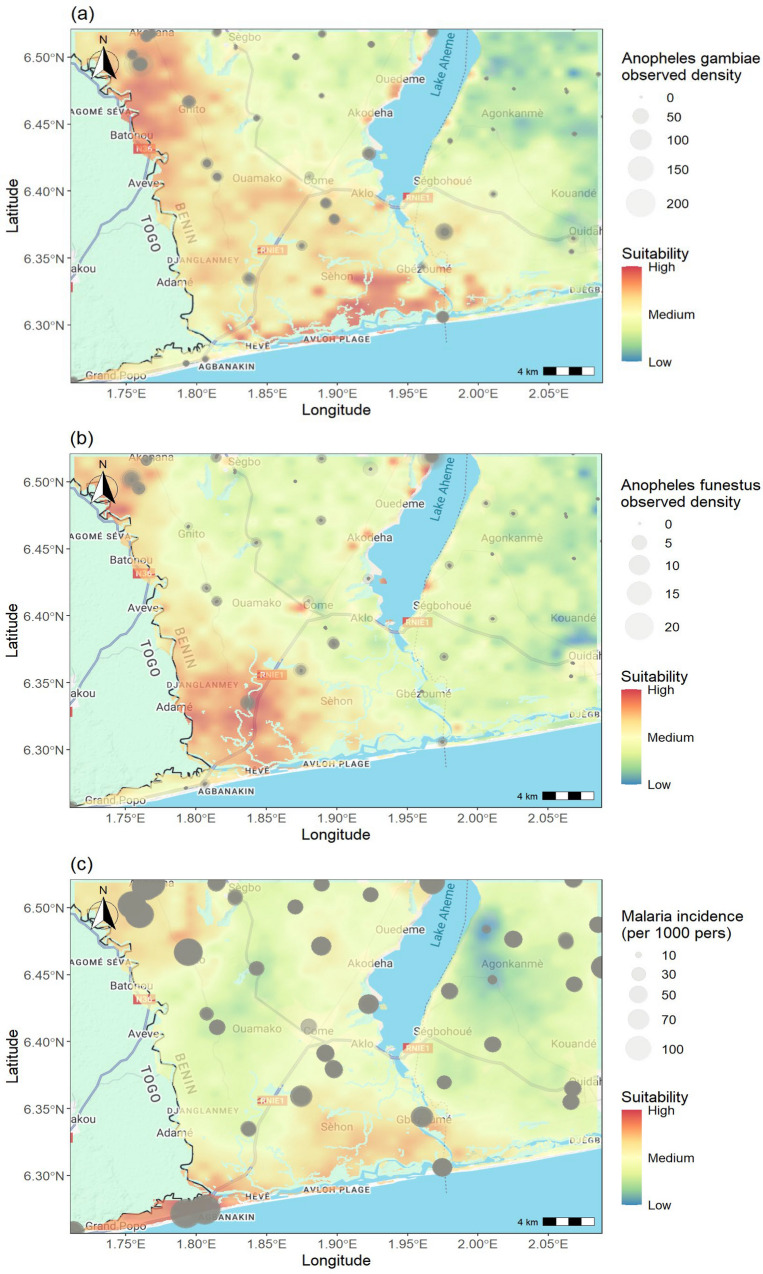
Suitability maps for *An. gambiae* (**a**), *An. funestus* (**b**) and Malaria incidence (**c**). Suitability maps are overlaid with observed data to assess spatial agreement between predicted suitability and actual data. Plot created with ‘ggmap’ and ‘ggplot2’ packages of R-cran software. Basemap source: Google Maps. Map data ©2025 Google

High suitability for *An. gambiae* is predominantly predicted in western part of the study area, particularly around Akonana in the northwest, and in Avloh district (Fig. 2a). These high suitability areas correspond closely with sites of elevated *An. gambiae* densities, as indicated by the larger gray circles. In contrast, eastern parts of the region display areas such as Kouandé and Ouidah with lower suitability (blue-green tones), and correspondingly lower observed densities.

Compared to *Anopheles gambiae,* predicted suitability is generally lower across the study area for *An. funestus*, with a few localized zones of elevated suitability (Fig. 2a, b). Notable highly suitable areas for this species are concentrated in the southwestern regions around Adjaha and Grand-Popo; and in northwestern areas around Akonana. Although *An. funestus* catches remain low overall, these areas highlighted in red correspond with slightly higher observed densities. On the contrary, most of the eastern regions display moderate to low suitability (green to blue tones), consistent with sparse vector observations.

For malaria incidence, predicted suitability is highest in northwestern and the southwestern coastal region of the study area, particularly around Grand-Popo, Agbanakin, and Gbézoumé, as indicated by the red shading (Fig. 2c). Those areas reported the largest observed incidence values, reflected by the large gray circles. Moderate suitability extends eastward toward Ségbohoué and Agonkanmè, while lower suitability is observed in the northeastern and central-western regions, where reported incidence is comparatively lower. However, in the south-eastern arrondissements, the predicted ecological suitability for malaria appeared moderate, while observed incidence was relatively low. These mismatches illustrate that ecological suitability does not always translate directly into epidemiological burden, as health system factors, including intervention coverage, access to healthcare, and reporting practices, can strongly influence the detection and recording of cases.

**Fig. 3:**
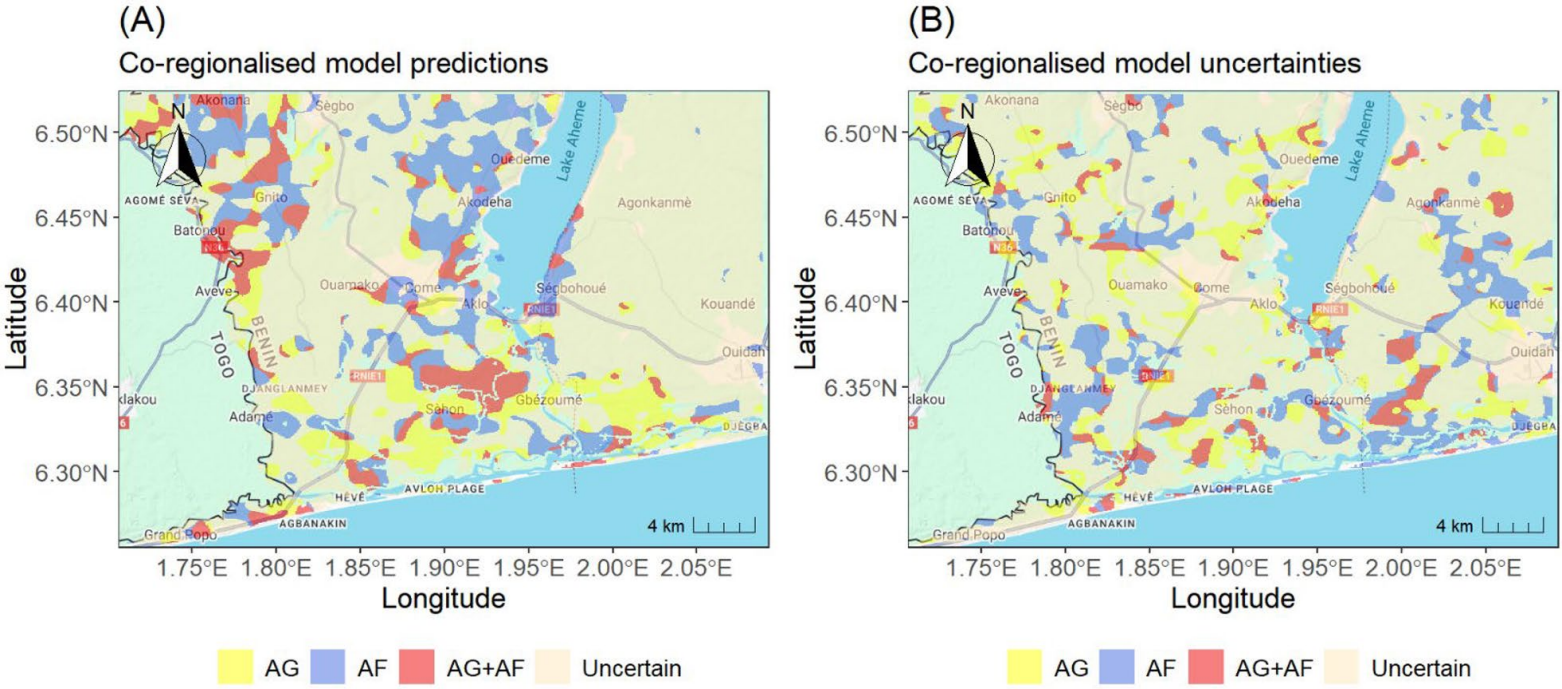
The co-regionalization analysis illustrates model *predictions* (**A**) and *uncertainties* (**B**). Different colors highlight areas where at least one Anopheles species (*An. gambiae* (AG) and *An. funestus* (AF)) and malaria suitability exceed 0.25, with a correlation between individual species and malaria above the 0.2. The map was generated using the ggmap and ggplot2 packages in R. Basemap source: Google Maps. Map data ©2025 Google

The co-regionalisation analysis identified distinct and spatially structured zones of joint suitability between malaria incidence and vector abundance. Areas of strongest joint association between malaria incidence and both *Anopheles gambiae* and *An. funestus* suitabilities (shown in red in Fig. 3A) form contiguous bands along the southern coastline, extending through Avloh Plage and inland toward the western shores of Lake Ahémé. Zones where malaria incidence is primarily associated with *An. gambiae* alone (yellow) are interspersed within these coastal clusters and occur as scattered inland patches, whereas areas with higher suitability of *An. funestus* (blue) are more widespread and are largely concentrated around lacustrine and wetland environments, particularly near Lake Ahémé. In contrast, uncertain areas (beige), where no clear co-regionalised association could be established, are more prevalent in the north-eastern part of the study area and appear as discontinuous patches elsewhere. Uncertainties in the vectors models are also positively correlated with malaria model uncertainty, but these associations are less geographically extended (Fig. 3A, B). These areas of high uncertainty correlation can likely be attributed to factors not considered in the analysis.

The computation of the land area associated with each type of vector–malaria co-suitability (Table 2) reveals that the largest proportion of the mapped area is significantly associated with *An. funestus* (AF), covering approximately 120 km^2^ (Table 2). Areas where both *An. gambiae* s.l. and *An. funestus* simultaneously exhibited high local correlation with malaria incidence (AG + AF) covered 67.3 km^2^, while areas associated primarily with *An. gambiae* s.l. (AG) spanned 89.9 km^2^. Regions where no clear spatial association was detected (classified as uncertain) accounted for 513 km^2^.

**Table 2:** Surface area of co-regionalised malaria–vector association types

Class label	Area (km^2^)	Areal proportion (%)
AG	89.900	11.38
AF	119.953	15.18
AG + AF	67.286	8.52
Uncertain	513.022	64.92

AG, *Anopheles gambiae* s.l; AF, *Anopheles funestus*

**Fig. 4:**
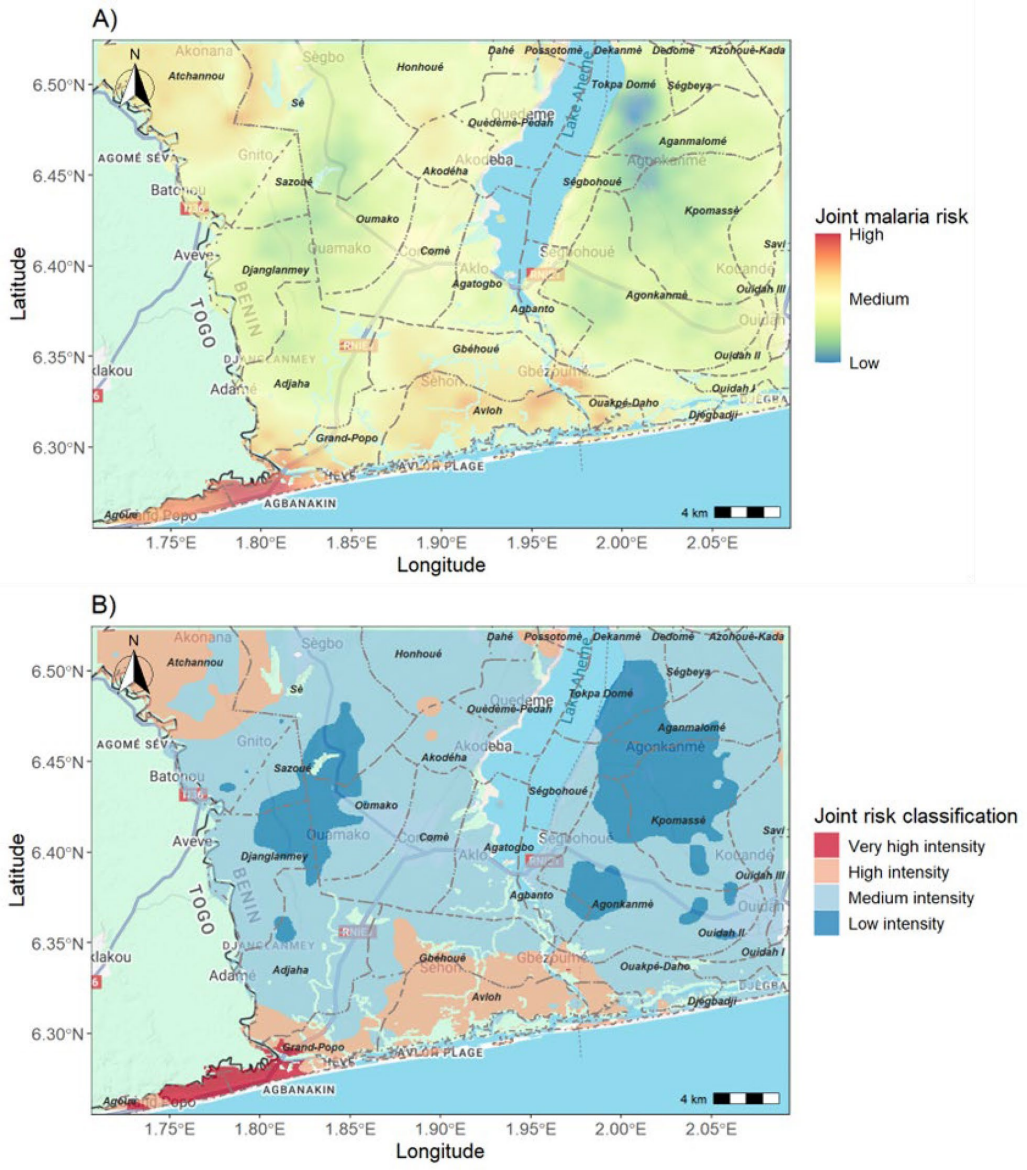
Maps of joint suitability to both malaria incidence and the two Anopheles species (**A**) and joint malaria risk classification (**B**). The joint malaria risk surface was normalized to a 0–1 scale and classified into four intensity categories using empirical thresholds: *very high* (≥ 0.8), *high* (≥ 0.6 to < 0.8), *medium* (≥ 0.3 to < 0.6), and *low* (< 0.3) Basemap source: Google Maps. Map data ©2025 Google

Very high malaria risk (in red on Fig. 4A) is predicted in southwest areas, namely in Agoue and Grand-Popo districts. In addition, large areas of high joint malaria risk (orange) are concentrated around Avloh Plage spreading inland towards the districts of Adjaha, Gbéhoué and Gbèzoumè, as well as in the northern districts of Possotomè, and Atchannou (Fig. 4B). Smaller pockets of these areas are also in Sè, Akodéha and Honhoué districts. In contrast, districts with the lowest median malaria risk are respectively Tokpa Domé, Kpomassè, Agonkanmè, Oumako, Djanglanmey, Ségbohoué, Aganmalomé, Sazoué, Sè, Ouidah II, and Ouidah I (see Table S4 in supplementary materials).

### Important environmental predictors

Out of 36 predictors considered in the variable’s selection process, eight were selected for *An. gambiae*, six for *An. funestus* and ten for malaria incidence (Table 3). Among these, three variables—maximum Leaf Area Index (LAI), wind speed (ws), and mean Mid-Infrared reflectance (MIR)—were common to all three processes. The latter environmental variable negatively influences all the three processes, suggesting that malaria incidence and vector abundance tend to increase in areas with lower MIR reflectance, a humidity indicator typically associated to vegetation moisture and surface wetness. Meanwhile, maximum leaf area index was associated with decreased malaria incidence and *An. gambiae *catches, but with increased *An. funestus* abundance, suggesting divergent ecological preferences between the two vector species. Similarly, wind speed emerged as a common predictor for both mosquitoes’ density and malaria incidence, but its effect varied in direction and strength. Wind speed had a strong negative association with *An. gambiae* catches, indicating that higher wind exposure may hinder the presence or activity of this species. In contrast, *An. funestus* abundance was positively associated with wind speed, suggesting that this species may be more tolerant of, or even benefit from, moderate wind conditions. Notably, wind speed also showed a negative association with malaria incidence, suggesting that higher wind exposure may disrupt mosquitoes host seeking or reduce biting rates.

**Table 3:** Mean and 95% credible interval of model parameters

Process	Model parameter	2.50%	Mean	97.50%
*Anopheles gambiae*	Intercept	− 2.849e + 00	− 6.805e-01	1.314e + 00
	evi	1.104e-04	1.996e-04	2.907e-04
	lai_max	− 5.226e-01	− 3.203e-01	− 1.075e-01
	dist_to_stag_water	4.156e-05	8.445e-05	1.296e-04
	lst_day_max	− 4.180e-03	3.718e-02	7.887e-02
	PET_avg	1.412e-03	2.846e-03	4.231e-03
	elevation	− 2.356e-02	− 1.806e-02	− 1.259e-02
	ws	− 6.716e-01	− 4.721e-01	− 2.627e-01
	mir_avg	− 3.359e-04	− 1.337e-04	6.097e-05
*Anopheles funestus*	Intercept	− 1.002e + 00	9.126e-01	2.851e + 00
	ws	1.423e-01	2.695e-01	3.846e-01
	lai_max	7.779e-02	1.362e-01	2.155e-01
	ppt	− 8.846e-05	2.108e-03	4.262e-03
	mir_avg	− 2.206e-04	− 1.442e-04	− 5.288e-05
	lst_night_min	− 4.581e-02	− 2.397e-02	− 8.495e-03
	aet	− 1.269e-02	− 5.237e-03	2.209e-03
Malaria incidence	Intercept	1.297e + 00	5.460e + 00	9.324e + 00
	anopheles_gambiae	− 8.456e-03	9.695e-03	2.385e-02
	dist_to_run_water	− 1.282e-04	− 3.269e-05	7.569e-05
	dist_to_stag_water	− 6.032e-05	3.364e-05	1.452e-04
	mir_avg	− 1.714e-04	− 1.018e-04	− 3.410e-05
	evi	− 8.656e-05	− 4.903e-05	− 1.875e-05
	lai_max	− 2.396e-01	1.297e-03	1.716e-01
	Landcover = Crops	− 1.451e-01	4.053e-02	2.043e-01
	Landcover = Built Areas	− 1.983e-01	− 3.221e-02	1.177e-01
	Landcover = Rangeland	− 2.361e-01	− 3.538e-02	1.182e-01
	hf_attendance_rate	− 4.977e-02	− 4.251e-02	− 3.559e-02
	lst_night_max	1.190e-02	3.030e-02	4.689e-02
	ws	2.472e-01	3.484e-01	4.393e-01
Joint parameters	Partial Sill or spatial variance	1.000e + 00	2.068e + 01	3.162e + 01
	Nugget effect	3.571e-02	9.154e-02	1.000e + 00
	Spatial range (degrees)	3.065e-01	3.065e-01	3.066e-01

evi, MODIS monthly Enhanced Vegetation Index; lai_max, maximum of MODIS 8-day composite leaf area index; dist_stag_water, distance to stagnant waters; lst_day_max, maximum of MODIS daily Daytime land surface temperature; PET_avg, average of MODIS 8-day composite Total Potential Evapotranspiration; ws, monthly weather station wind speed; mir_avg, average of MODIS mid-infrared reflectance; ppt, monthly precipitation from TerraClimate; lst_night_min, minimum of MODIS daily nighttime land surface temperature; aet, TerraClimate monthly actual evapotranspiration; *Anopheles*_gambiae, density of *Anopheles gambiae* mosquitoes; dist_to_run_water, distance to running waters; lst_night_max, maximum of MODIS daily nighttime land surface temperature; hf_attendance_rate, health facilities’ attendance rate

Monthly Enhanced Vegetation Index (EVI) and distance to stagnant water were shared predictors of both malaria incidence and *An. gambiae* abundance, though their effects were antagonistic—positively influencing the latter while negatively affecting malaria incidence. Apart from these common predictors, the three models retained distinct sets of important predictors, highlighting differences in the spatial distribution and ecological preferences of *Anopheles gambiae*, *An. funestus*, and malaria incidence. Different forms of land surface temperature notably emerged as environmental drivers across the three processes: for *An. gambiae*, maximum daytime land surface temperature (LST_day_max) was positively associated with mosquito abundance, while malaria incidence was positively associated with maximum nighttime land surface temperature (LST_night_max). In contrast, *An. funestus* abundance was negatively associated with minimum nighttime land surface temperature (LST_night_min), suggesting differing behaviour or thermal tolerances among the two vector species and their contribution to malaria transmission dynamics.

Besides land surface temperature, *An. gambiae* catches were positively associated with average potential evapotranspiration. In contrast, increasing elevation and average mid-infrared reflectance limit *An. gambiae* abundance. Malaria incidence showed a weak positive association with *An. gambiae* density, while being negatively influenced by distance to running water, though with high prediction uncertainty. All vegetation-related predictors (EVI, and LAI) showed negative effects on malaria incidence, potentially due to densely vegetated constraints on mosquito breeding or reduced human-vector contact in densely vegetated landscapes. Health facility attendance rate (hf_attendance_rate) was negatively associated with malaria incidence (mean =  − 0.0425; 95% credible interval: − 0.0498 to − 0.0356). This suggests that higher access to or utilization of health services may have diluted reported malaria cases.

### The common spatial process for vectors and malaria

The joint model for *Anopheles gambiae*, *Anopheles funestus*, and malaria incidence shared a common spatial structure which had a range parameter (*φ*) of 0.306 degrees. It defines the distance at which spatial correlation decays, which corresponds to around 34 km (Table 3). The proportion of spatial autocorrelation can be inferred from the nugget-to-sill ratio (Cressie, 2015). In this study, the proportion of pure error (nugget effect, *τ*^2^) relative to the total variance (partial sill + nugget effect) is 0.44%.

### Model evaluation and validation

A large proportion of the variation in malaria incidence data was explained by the predictors (96,1%), as opposed to approximately one-third of the variance explained for *An. gambiae* and *An. funestus* (Table 4). That high proportion of explained variance by the fixed effect does not mean a poorly fitted random effect, but indicates that the selected predictors accounted for most of the observed variability in malaria incidence. Within the joint modelling framework, the *An. gambiae* model shows the greatest predictive uncertainty, with a mean error of 0.306, the largest residual variance (MSE = 0.98), and root mean squared error of 0.83. However, its 94.5% predictive-interval coverage is the closest of the three models to the ideal 95%, indicating well-calibrated uncertainty. The *An. funestus* model has a mean error of −0.16, the smallest residual variance (MSE = 0.18), and RMSE of 0.40.

**Table 4:** Model evaluation (ME and MSE) and validation (Coverage and RMSE) statistics

Process	ME	MSE	COV (%)	RMSE	Explained variance by the fixed effect (%)
*An. gambiae*	0.306	0.983	94.505	0.825	30.648
*An. funestus*	− 0.164	0.177	93.407	0.393	35.349
Malaria incidence	− 0.470	0.742	87.302	0.260	96.097

The malaria incidence model shows systematic underprediction (ME = −0.47), but achieves relatively low overall prediction error (RMSE = 0.26) once both bias and variability are taken into account. The high proportion of variance explained by fixed effects indicates that the selected covariates captured a substantial share of the spatial variability in malaria incidence. It should be noted that ME and RMSE are scale-dependent metrics and therefore cannot be directly compared across the three models, which are fitted to outcomes measured on different scales (Table 4).

## Discussion

Identifying malaria hotspots is crucial for effective control planning and disease elimination efforts, as it facilitates the understanding and monitoring of the transmission patterns in these areas characterized by higher malaria incidence compared to neighbouring areas [18]; in particular, it supports the design of evidence-based interventions tailored to specific malaria-endemic regions [17]. Hotspots are theoretically acknowledged as the geographical evidence of the existence of heterogeneity in malaria distribution [26]. Although the literature highlights heterogeneity in malaria transmission in Benin, to our knowledge, no studies have provided an empirical description of epidemiological and entomological malaria hotspots in the study area. The current work is the first to achieve this in southwestern Benin, using a joint spatial modelling framework that leverages our current knowledge of the ecological distribution of two *Anopheles* mosquitoes, primary vectors for malaria, and the epidemiological context of malaria in this region.

### Our definition of malaria hotspot

In our study, malaria hotspots are defined as areas with higher-than-average risk of malaria incidence, as identified by a model-based geostatistical approach that thresholds a joint malaria risk surface defined as the product of predicted malaria incidence and the probability of presence of both *Anopheles gambiae* and *Anopheles funestus*. This choice is subjective since no strict definition of malaria hotspots exists. Malaria hotspots have been defined statistically [45] from the analysis of a diverse range of indicators [17]. These metrics include but are not limited to mosquito flight behaviour [16], mosquito abundance [18], exposure to infected mosquitoes [46], micro-epidemiological elevations in malaria incidence [46–48], asymptomatic parasite carriage [47, 48], prevalence of *Plasmodium* parasites in the human population [47], and serological responses to malaria-specific antigens [49]. All the described methods identify local anomalies compared to a given benchmark, and do not attempt to map the different intensities of hotspots along the region. This is the novelty of our definition.

### Configuration and interpretations of identified malaria hotspots

The malaria hotspots identified in this study are contiguous high-risk zones ranging from 4 to 20 km^2^ to entire blocks of arrondissements (see red or orange zones in Fig. 4B). These hotspots differ in configuration from the traditional notion of malaria hotspots, as entire sub-national administrative units with above-average incidence. Instead, they reflect a more nuanced understanding of malaria heterogeneity, where nearby localities within the lowest sub-national administrative unit can experience very different levels of risk and burden—differences that are often missed by routine disease surveillance systems [50].

Our results support other findings suggesting that the size and boundaries of malaria hotspots can depend significantly on the analysis tools and metrics used for their identification [49]. Compared to studies in sub-Saharan Africa that rely on traditional cluster detection algorithms [51], the hotspots identified by our joint modelling differ notably in both configuration and scale, extending well beyond the typical hotspot cluster size of 1 km^2^ [47]. The size of hotspots identified in this study also differs from those reported in other studies applying model-based geostatistical methods. For instance, Bayode and Siegmund [52] mapped under-five malaria prevalence in Akure, Nigeria, using 100 × 100 m resolution grids and identified hotspots (≥ 10% prevalence) that were spatially more confined, likely due to the high granularity of the data. In Kenya, using a coarser 5 × 5 km resolution, revealed broader hotspots concentrated around the Lake Victoria endemic region [53]. Our results align more closely with findings of [54] in Ghana, where Bayesian spatiotemporal modelling revealed expansive regions of elevated malaria risk among children under five. The identification of such large-scale malaria hotspots in our study is attributable to three key factors: the quite broad geographic extent of our analysis, which allowed us to detect spatially coherent risk patterns across diverse ecological zones; the joint modelling of high-resolution entomological and epidemiological data, which when aggregated across broad geographic areas, revealed consistent large-scale spatial patterns between vector abundance and malaria incidence; and the use of multi-scale environmental predictors, which enabled the detection of regional climatic and ecological drivers that influence malaria transmission dynamics across extensive geographic areas.

The high malaria risk observed in the predicted hotspots is likely driven by a complex interplay of entomological, environmental, and anthropogenic factors. In southern Benin, research has established that the expansion of irrigated vegetable farming, rice cultivation, and inland fisheries has increased the availability of standing water bodies, ideal breeding sites for malaria vectors, particularly *An. gambiae* [55]. These agricultural landscapes not only extend the duration of aquatic habitats but also alter local microclimates, favouring year-round vector survival and proliferation. This is confirmed by the importance of MIR that was found for both mosquitoes and malaria.

The use of agricultural pesticides has contributed to the emergence and spread of insecticide resistance in mosquito populations. Numerous studies have documented high levels of resistance to pyrethroids and carbamates among both *An. gambiae* and *An. funestus* across all regions of Benin, which undermines the effectiveness of long-lasting insecticidal nets (LLINs) and indoor residual spraying (IRS) [56, 57]. In hotspot areas, this resistance may allow vector populations to persist despite high intervention coverage, contributing to residual transmission.

In addition, disparities in use of healthcare services, as suggested by the negative association between health facility attendance and malaria incidence, may exacerbate vulnerability in ecologically suitable zones. Taken together, these findings underscore the need for future integrated malaria control strategies to consider the ecology, human behaviour and service accessibility in spatially targeted interventions.

It is important to note that our data collection was limited to the dry season, which carries significant implications for the temporal interpretation of the identified hotspots. Malaria transmission is known to exhibit strong seasonal variation in many parts of sub-Saharan Africa, including southern Benin, where peak transmission typically coincides with the rainy season due to increased vector breeding sites [56]. As such, hotspots reflect areas of sustained transmission during the typically low-transmission period. This suggests that these zones may represent persistent or dry-season hotspots—locations where transmission continues even when vector densities and overall incidence are expected to decline [26]. Studies have shown that such areas often play a key role in sustaining malaria transmission across seasons and may seed infections during the rainy season [19]. However, the temporal stability of hotspots is not guaranteed; hotspots identified in one season may dissipate or shift in another, especially under changing environmental, entomological, or anthropogenic conditions [47]. Consequently, our findings should be interpreted as a description of time-specific malaria transmission patterns, and further investigation with longitudinal, multi-seasonal data is needed to assess the persistence and recurrence of these hotspots.

### Advantages of our modelling framework

Jointly modelling mosquito abundance and malaria incidence within a model-based geostatistical framework offers several advantages. It captures complex interactions between entomological and epidemiological processes, enabling a nuanced understanding of how vector dynamics shape malaria transmission. It also improves predictive accuracy [6], by accounting for spatial correlations between vector abundance and disease outcomes. Moreover, the correlations estimated between outcomes within the joint model are less biased than those derived from independent modelling [6]. Operationally, this approach can facilitate more effective targeting of interventions by pinpointing areas where high mosquito density aligns with elevated malaria incidence, thereby offering an opportunity to optimize resource allocation. It also supports evidence-based decision-making by providing granular, spatially explicit insights for prioritizing control efforts.

### Insight into the co-regionalized transmission patterns

The coregionalization analysis revealed that the spatially correlated suitabilities between vectors and malaria data covered a large portion of the region (Fig. 3A). In comparison to *An. gambiae,* the correlated suitability of *An. funestus* with malaria incidence covers a larger area (Table 2). This aligns well with recent evidence of *An. funestus* to be widely distributed across sub-Saharan Africa [58, 59], and dominant in most settings in east and southern Africa [60]. However, *Anopheles gambiae* counts has the strongest direct association with malaria because it was selected as predictor for malaria incidence. These results are not contradicting each other, since the suitability maps account for both risk factors and spatio-temporal trends, the latter is likely driven the higher geographical association between malaria and *Anopheles funestus* suitabilities. This finding has important consequence for malaria control since *Anopheles funestus* is highly resistant to common insecticides used by public health authorities [61].

The surface area estimates provided in Table 2 also reveals areas where both species co-regionalized, highlighting locations of potentially synergistic transmission dynamics. Such areas, dominated by overlapping vector–malaria associations, may challenge the effectiveness of species-specific interventions and increase the risk of control failure if one vector is overlooked [62].

The map of co-regionalized uncertainties (Fig. 3B) reveals several areas where unexplained variation in vector and malaria suitability aligns. These zones may reflect either model uncertainties or the influence of unmeasured predictors affecting both vector and malaria suitability, including socio-economical and behavioural factors such as human mobility, housing quality, and intervention coverage [6]. Further investigation of these areas can offer an opportunity to enhance surveillance and improve malaria risk mapping in the region; and to uncover hidden drivers of transmission risk [50].

The observation of high malaria incidence in areas where observed mosquito density is low, such as Grand-Popo (Fig. 2a–c), does not necessarily imply a failure of the joint modelling framework. Malaria incidence reflects cumulative transmission processes that may be influenced by temporal lags due to climatic drivers [63], human mobility [64], healthcare access, and reporting practices [65], while entomological data provide sparse, time-specific snapshots of vector abundance. The shared spatial component of the model is therefore not designed to enforce strict local agreement between mosquito counts and malaria incidence, but rather to capture underlying spatial drivers common to both processes that are not directly measured. From this perspective, areas with high incidence but low observed vector density likely reflect residual transmission driven by unobserved or temporally decoupled mechanisms rather than model misspecification.

### Environmental drivers of vector abundance and malaria incidence

Our analysis also identified some important environmental drivers of *An. gambiae*, *An. funestus* abundance, and malaria incidence in southern Benin, highlighting species-specific ecological preferences and shared landscape influences. Three key environmental variables were revealed as consistent predictors of both malaria incidence and vector abundance such as: Mid-Infrared Reflectance (MIR), Leaf Area Index (LAI), and wind speed. Lower MIR values, typically associated with moist, densely vegetated, or shaded environments [66], were consistently associated with increased suitability for both vectors and malaria, aligning with findings that MIR captures soil and vegetation moisture crucial for sustaining *Anopheles* breeding habitats and adult survival [67]. LAI exhibited divergent associations with the two malaria vectors, reflecting their distinct ecological requirements. Increased LAI was linked to reduced abundance of *An. gambiae* and lower malaria incidence, whereas it positively correlated with higher densities of *An. funestus*. This aligns with findings from previous studies in Southern Benin by Cottrell and colleagues [68], who reported that *An. gambiae* primarily thrives in environments with low-to-moderate vegetation cover. Similarly, Kahamba and colleagues investigated *An. funestus* breeding in southeastern Tanzania and confirmed that this species thrives in permanent, shaded water bodies with emergent vegetation, where larval occurrence is strongly linked to dense tree cover [69]. Wind speed also exhibited opposite associations, limiting to *An. gambiae* while being more tolerated by *An. funestus*, and reducing malaria risk overall. These observations are partially supported by the findings of other studies that describe the key role of wind upon the suitability of *Anopheles* species [6]*,* but in line with modelling studies showing that wind can disrupt mosquito flight and therefore malaria transmission [70].

Additional environmental variables, such as the Enhanced Vegetation Index (EVI), land surface temperature (LST), and potential evapotranspiration (PET), showed distinct associations. Adult mosquito survival and biting frequency vary with air temperatures [71]. In fact, both species have specific temperature preferences [72] and their distinct behavioural patterns: *An. gambiae* s.l. is predominantly a nighttime biter [73] with exophagic (outdoor-biting) and endophilic (indoor-resting) behaviour [74, 75]. Then, higher daytime temperatures during the dry season increase indoor catches as humans spend more time outdoors, enhancing mosquito-human contact, after which mosquitoes rest indoors where sampling occurred. While also being anthropophilic, nightime bitter, and endophilic (indoor-resting), *An. funestus* exhibits both exophagic (outdoor-biting) and endophagic behaviour, with frequent blood-feeding patterns often requiring multiple blood meals before laying eggs [76]. Hence, lower nighttime temperatures which reduce human outdoor activity during these critical feeding periods, can limit this species contacts opportunities with humans that are essential for *An. funestus*' frequent blood-feeding requirements and subsequently constraining population growth through reduced reproductive success.

Vegetation structure, as captured by EVI, also revealed divergent associations. While EVI positively influenced *An. gambiae* abundance—likely by sustaining moderate larval habitats—it showed a negative association with malaria incidence, perhaps due to reduced human-vector contact in densely vegetated areas or lower population density [77]. Similarly, potential evapotranspiration was positively associated with *An. gambiae*, indicating this species’ preference for warmer, open environments [78], while *An. funestus* was more common in areas with lower evapotranspiration, consistent with its affinity for stable, shaded aquatic habitats [79]. Proximity to stagnant water correlated positively with *An. gambiae* abundance but negatively with malaria incidence, possibly due to local variations in human settlement patterns or mosquito avoidance behaviours. Similarly, distance to running water showed a negative association with malaria incidence. Taken together, the observed effects with these variables reflecting distance to mosquitoes’ breeding site support that hydrological factors may influence human–vector interactions beyond larval site availability [26]. Finally, greater attendance to health facilities was strongly associated with reduced malaria incidence, underscoring the protective effect of improved use of healthcare services, even in areas environmentally conducive to high malaria transmission. This may also reflect the common practice in endemic settings of presumptively treating febrile illness as malaria, which can reduce the infectious reservoir and interrupt transmission [80].

### Implications for targeted malaria control

This study provides valuable insights into where intensified malaria control efforts could reduce transmission intensity in the community as a whole. The identified hotspots—spatially contiguous areas where both malaria incidence and vector abundance suitabilities are correlated and the highest—offer a practical entry point for subnational tailoring (SNT) of interventions, as recently encouraged by WHO and partners [81]. These zones span multiples villages and ecological contexts, underlining the need to move beyond administrative boundaries when planning interventions.

In areas where both *An. gambiae* and *An. funestus* occur and their distribution are jointly correlated with malaria cases is crucial to adopt species-agnostic strategies. These could include indoor residual spraying with non-pyrethroid insecticides, larval source management tailored to local aquatic habitats, and community-level vector surveillance. Given the growing resistance to conventional insecticides observed in southern Benin [56, 57]these strategies must be informed by up-to-date resistance profiles and vector behaviour.

For people living in the identified high-risk areas, especially where low health facility attendance is observed, targeted case detection and treatment campaigns—especially during the dry season when transmission persists—can help reduce the human reservoir [17, 82]. Outreach campaigns should also be organized in those regions to address care-seeking behaviour and treatment delays, both of which may contribute to sustained transmission. Efforts to better protect populations living in hotspots should also include the adoption of next-generation dual-active ingredient nets, rotational indoor residual spraying with non-pyrethroid insecticides, and continuous resistance monitoring tailored to hotspot areas [83].

### Limitations and recommendation for future research

This work has some limitations. First, the vector data used in our analysis were only available for two time points (October and November 2018 and 2021). That may have contributed to the high spatial heterogeneity in mosquitoes’ data and affected the robustness of the variable selection process. It also limits the expansion of hotspots delineation to others time periods, including the high malaria transmission seasons.

Within our modelling framework, we log-transformed counts of *An. gambiae* and *An. funestus*, and malaria incidence, rather than modelling them using Poisson or negative binomial likelihoods with a log link. Although count-based distributions are commonly used for entomological data, we adopted a joint log-Gaussian framework to stabilise variance and to ensure computational efficiency. Log-transformation of insect abundance data has been widely used in ecological and spatial epidemiological studies to enable inference within Gaussian spatial processes, particularly when multivariate or joint spatial structures are required [84–87]. Our use of log-transformed outcomes was therefore a pragmatic modelling choice to ensure tractability within a joint spatial framework with a common spatial random effect, rather than a rejection of count-based likelihoods in principle.

Malaria incidence data were available by arrondissement only, which is the lowest administrative level for which malaria case data are available in the DHIS2 [88]. Similarly, malaria cases were provided monthly as opposed to vector data collected weekly. In addition, population data necessary to compute malaria incidence is provided for the full year rather than for the specific period of mosquito surveillance. It is likely, therefore, that the significant component of uncertainties has been inflated by the different spatial and temporal resolutions of the datasets.

We acknowledge that using mosquito abundance alone as a proxy for malaria transmission intensity presents limitations. While vector density provides essential information on potential exposure to malaria, it does not distinguish between infected and non-infected mosquitoes or account for variability in human–vector contact. More informative entomological indicators, such as the entomological inoculation rate (EIR), human biting rate, or sporozoite rate, could provide a better and direct measure of transmission potential. Incorporating these entomological metrics would refine malaria hotspot mapping, better inform intervention prioritization, and strengthen validity of entomological–epidemiological links. Future efforts should aim to collect and integrate these complementary data streams where feasible into spatial models to enhance their predictive performance and operational utility.

This study focused on estimating the shared spatial structure among entomological and epidemiological processes, with efforts made to reduce variance-related biases by incorporating environmental and climatic covariates at finer resolution than the response variables—an approach supported in previous work [89]. While the spatial extent of our analysis is relatively small, the ecological and epidemiological context is representative of much of southwestern Benin, suggesting that findings may be relevant for similar coastal or peri-urban malaria-endemic settings. However, spatial models are known to be sensitive to local factors (such as human behaviour, mobility patterns, land-use fragmentation, and intervention history), thus limiting the generalizability of results beyond ecological interpretations, and ecologically similar areas. The spatial range estimated in this study (approximately 34 km) exceeds typical mosquito flight distances [16], possibly capturing unmeasured processes influencing both vector presence and malaria burden. The limited set of environmental covariates used may have led to an overestimation of spatial random effects. These limitations highlight the need for follow-up studies with expanded spatial coverage and resolution, higher temporal resolution, and additional explanatory factors such socio-economic and human mobility predictors. Finally, the largest share of the mapped area (513.022 km^2^) falls under the “Uncertain” category, where the association between malaria and vector suitability did not meet the correlation threshold—likely reflecting the limits of the input data.

## Supplementary Information

Additional file 1.

## Data Availability

Data used for the joint spatial model are available under restricted access for editorial and peer-review purposes: (https://zenodo.org/records/17296379). Model code can be found here: (https://zenodo.org/records/10258689).
