# Supplementary material for: Identification of malaria hotspots in southwestern Benin through spatial joint modelling of malaria incidence and vector abundance
Source: Malar J. 2026 Apr 4;25:219. doi: 10.1186/s12936-026-05891-3 (PMC13188395; doi:10.1186/s12936-026-05891-3)
Supplement: Supplementary file 1 — Additional file 1. [file 12936_2026_5891_MOESM1_ESM.docx]

**Supplementary materials**

**Table S1**: Explanatory variables data sources and properties

| Variable | Source | Period covered | Unit | Spatial resolution | Temporal resolution |
| --- | --- | --- | --- | --- | --- |
| soil texture at depth 0-5 cm | [ISRIC - World Soil Information](https://data.isric.org/geonetwork/srv/eng/catalog.search#/metadata/2a7d2fb8-e0db-4a4b-9661-4809865aaccf) | static | - | 250 m | - |
| soil drainage at depth | [ISRIC - World Soil Information](https://data.isric.org/geonetwork/srv/eng/catalog.search#/metadata/953d0964-6746-489a-a8d1-f188595516a9) | static | - | 250 m | - |
| landcover | [Esri Land Cover - ArcGIS Living Atlas](https://livingatlas.arcgis.com/landcover/) | 2017-2023 | - | 10 m | Yearly |
| leaf vegetation index | [MOD15A2H](https://lpdaac.usgs.gov/products/mod15a2hv006/) from [NASA Earthdata Search](https://search.earthdata.nasa.gov/search) | 2000 to the near-present | m²/m² | 500 m | 8-day |
| Net evapotranspiration | [MOD16A2GF](https://lpdaac.usgs.gov/products/mod16a2gfv061/) from [NASA Earthdata Search](https://search.earthdata.nasa.gov/search) | 2000 to the near-present | kg/m²/8day | 500 m | 8-day |
| - actual evapotranspiration [aet] (mm) - precipitation [ppt](mm) - wind speed [ws] (m/s) - soil moisture [soil] (mm) - maximum temperature aka [tmax] (°C) - minimum temperature [tmin] (°C) - runoff [q] (mm) | [TerraClimate](https://www.climatologylab.org/terraclimate.html) | 1958 to near-present | - | ~ 4km | Monthly |
| - vegetation indices: EVI and MIR reflectance | [MOD13A2](https://lpdaac.usgs.gov/products/mod13a2v061/) from [NASA Earthdata Search](https://search.earthdata.nasa.gov/search) | 2000 to near-present | [check valid ranges](https://www.ctahr.hawaii.edu/grem/mod13ug/sect0007.html) here | 1km | 16-Day |
| - vegetation indices: EVI and MIR reflectance | [MOD13A3](https://lpdaac.usgs.gov/products/mod13a3v061/) from [NASA Earthdata Search](https://search.earthdata.nasa.gov/search?q=MOD13A3) | 2000 to near-present | [check valid ranges](https://www.ctahr.hawaii.edu/grem/mod13ug/sect0006.html) here | 1 km | monthly |
| Elevation (DEM) | [ASTER Global Digital Elevation Model (ASTGTM)](https://search.earthdata.nasa.gov/search?q=ASTGTM) | static | meters above sea level | 30 m | - |
| - slope | Derived from DEM data from ASTGTM | static | degrees | 30 m | - |
| - distance to running waters - distance to stagnant waters | Derived from shapefiles of waterways in Benin downloaded from [OSM database](https://download.geofabrik.de/africa.html) | static | meters | ~ 1 km | - |
| MODIS Land Surface Temperature and Emissivity (MOD11A1) | NASA Earthdata Search | 2000 to near-present | Kelvin (for temperature), unitless (for emissivity) | 1 km | Daily |
| precipitation | USGS FEWS NET Data Portal | 1981 to near-present | millimetres (mm) | ~ 5.3 km | monthly |
| evapotranspiration | USGS FEWS NET Data Portal | 2012 to present | millimetres (mm | 1 km | monthly |


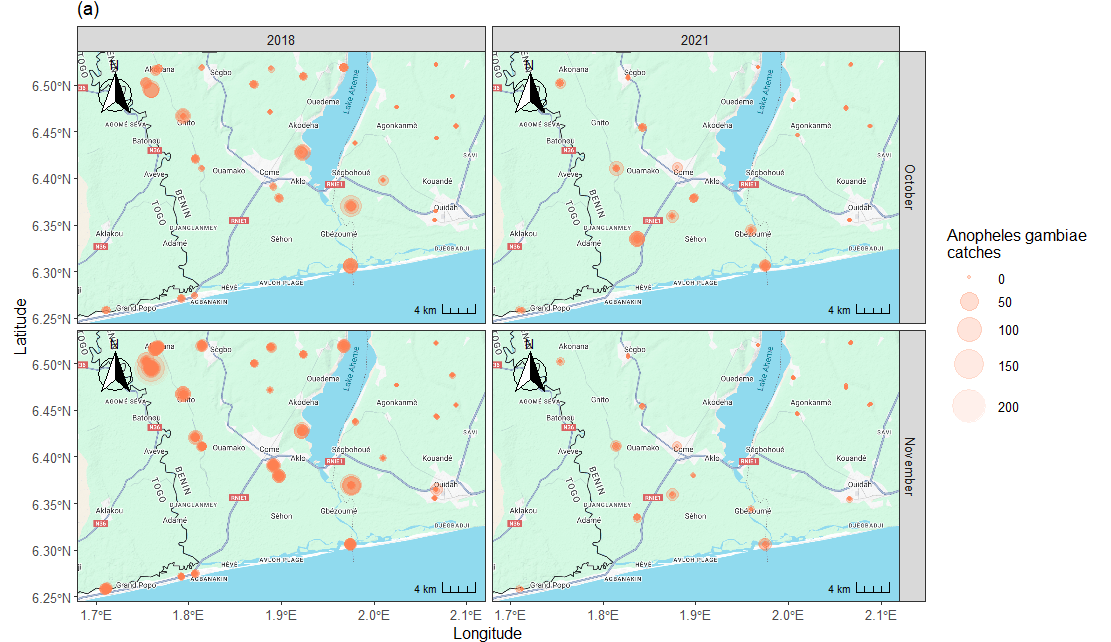

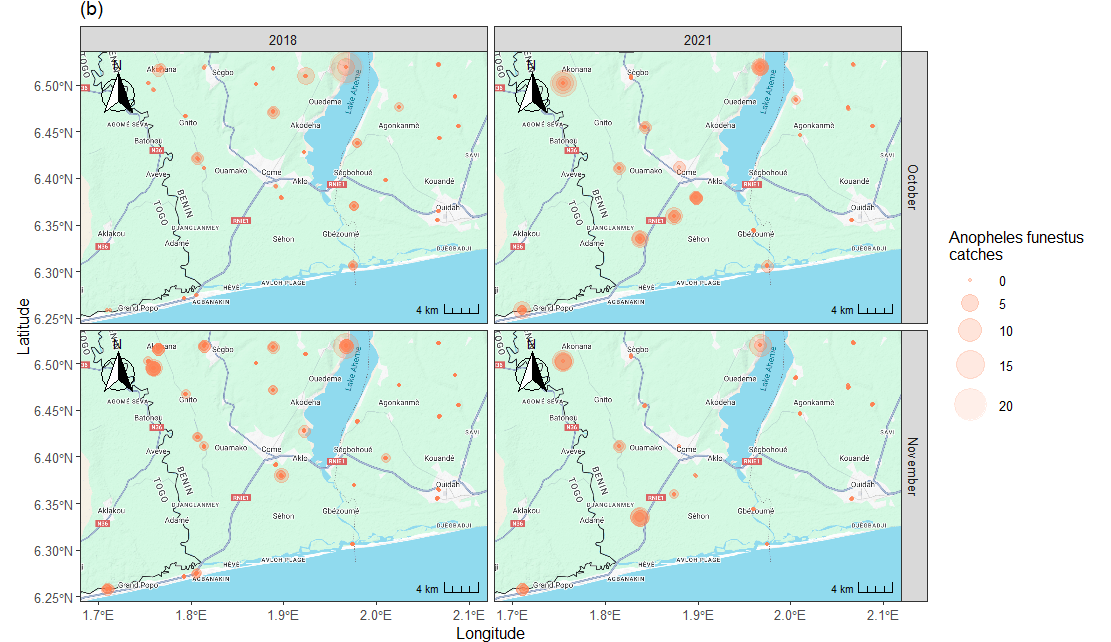

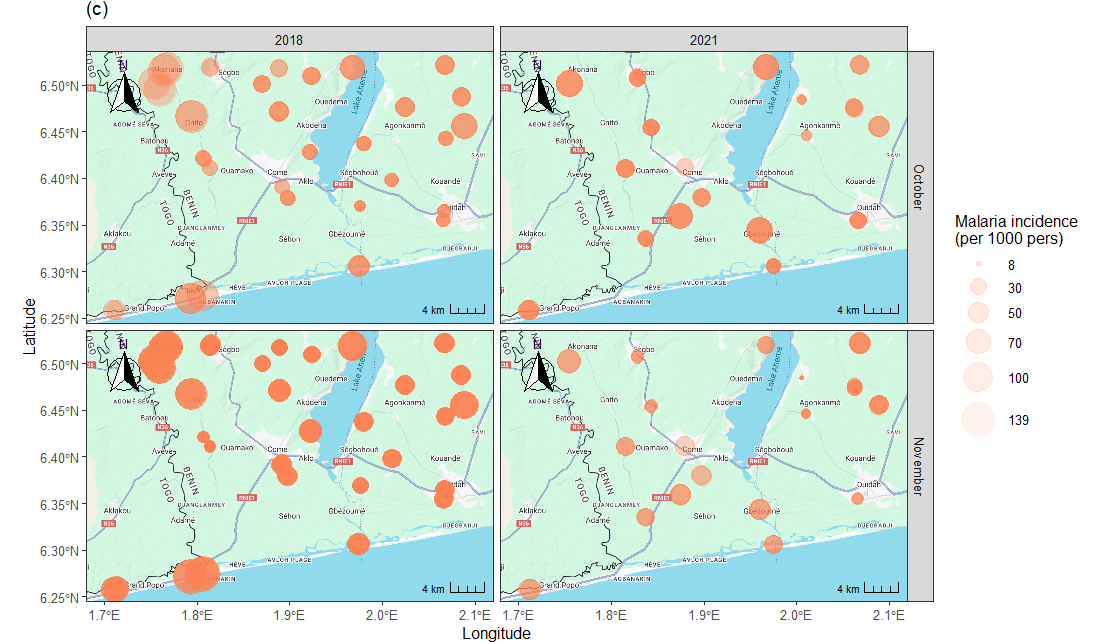


**Figure S2**: Spatiotemporal distribution of malaria vectors (female mosquitoes) and malaria incidence across villages in the study area. (a) *An. gambiae* catches, (b) *An. funestus* catches, and (c) malaria incidence per 1,000 persons, are shown by village for October and November in 2018 and 2021 Circle sizes and color intensities reflect catch abundance or incidence magnitude. Basemap source: Google Maps. Map data ©2025 Google.


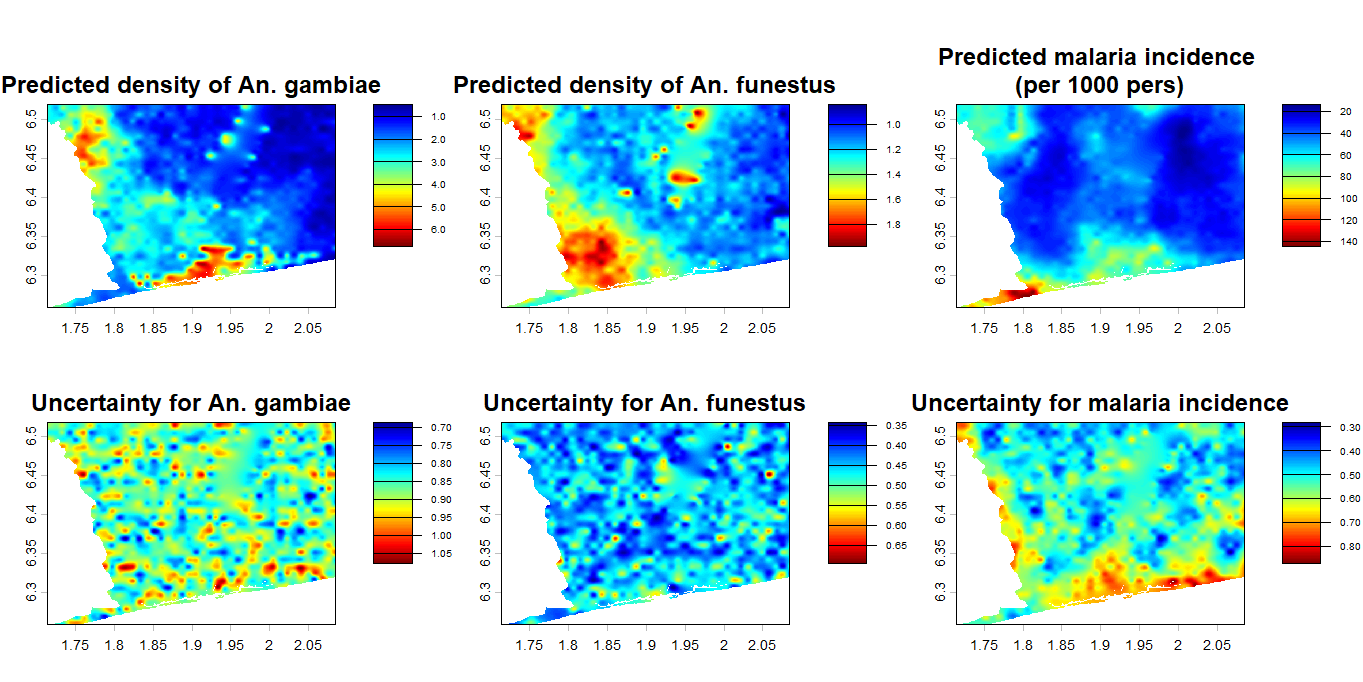


**Figure S3**: Spatial patterns of mean (top row) and the standard deviation (bottom row) of the predicted posterior distribution for mosquito densities and malaria incidence.

**Table S4**: Arrondissements ranking based on joint risk of malaria incidence, and abundance of *Anopheles gambiae* s.l. and *Anopheles funestus*.

| **Department** | **Province** | **District** | **Median risk** | **Rank** |
| --- | --- | --- | --- | --- |
| Mono | Grand-Popo | Agoue | 0.85204446 | 1 |
| Mono | Bopa | Possotomè | 0.66782377 | 2 |
| Mono | Grand-Popo | Avloh | 0.66646507 | 3 |
| Mono | Grand-Popo | Grand-Popo | 0.62552804 | 4 |
| Mono | Athiémè | Atchannou | 0.60012712 | 5 |
| Atlantique | Ouidah | Djégbadji | 0.59161022 | 6 |
| Mono | Grand-Popo | Gbéhoué | 0.58083975 | 7 |
| Atlantique | Tori-Bossito | Azohouè-Kada | 0.54317136 | 8 |
| Mono | Comè | Akodéha | 0.53452233 | 9 |
| Atlantique | Tori-Bossito | Bossito | 0.52321051 | 10 |
| Atlantique | Ouidah | Ouakpé-Daho | 0.51934986 | 11 |
| Mono | Houéyogbé | Dahé | 0.51519772 | 12 |
| Atlantique | Kpomassè | Dedomè | 0.48468726 | 13 |
| Mono | Comè | Ouèdèmè-Pédah | 0.48152661 | 14 |
| Mono | Houéyogbé | Honhoué | 0.46178889 | 15 |
| Mono | Comè | Comè | 0.46136998 | 16 |
| Mono | Comè | Agatogbo | 0.45021611 | 17 |
| Atlantique | Kpomassè | Agbanto | 0.44522888 | 18 |
| Atlantique | Ouidah | Ouidah III | 0.42763563 | 19 |
| Atlantique | Ouidah | Savi | 0.4203688 | 20 |
| Atlantique | Kpomassè | Ségbeya | 0.41748536 | 21 |
| Atlantique | Kpomassè | Dekanmè | 0.41387116 | 22 |
| Mono | Grand-Popo | Adjaha | 0.39737304 | 23 |
| Atlantique | Ouidah | Ouidah I | 0.39517291 | 24 |
| Atlantique | Ouidah | Ouidah II | 0.37743586 | 25 |
| Mono | Houéyogbé | Sè | 0.37729766 | 26 |
| Mono | Grand-Popo | Sazoué | 0.36954668 | 27 |
| Atlantique | Kpomassè | Aganmalomé | 0.36805756 | 28 |
| Atlantique | Kpomassè | Ségbohoué | 0.34350553 | 29 |
| Mono | Grand-Popo | Djanglanmey | 0.34041607 | 30 |
| Mono | Comè | Oumako | 0.33638025 | 31 |
| Atlantique | Kpomassè | Agonkanmè | 0.33349543 | 32 |
| Atlantique | Kpomassè | Kpomassè | 0.28582732 | 33 |
| Atlantique | Kpomassè | Tokpa Domé | 0.23272433 | 34 |

**Supplementary Information S5: Full description of the multivariate model**

Using vectorized notation for a spatial model with nugget effects, the multivariate model can be expressed as:

$$\log\left( y_{j} \right)\boldsymbol{|} \omega\sim N (\boldsymbol{X}_{j}\beta_{j}+ \omega, \varepsilon)$$

With $\omega\sim N (0, \sigma^{2}\boldsymbol{H})$ ; and $\varepsilon\sim N (0, \tau^{2}\boldsymbol{I})$

Here ***X*** represents a design matrix of predictors values, β represents the unknown regression coefficients. *ω* is a zero-mean Gaussian process with an exponential covariance function ***H*** characterized by unknown parameters common to all models, and spatially dependent variance *σ ^2^*. *ε* is the nugget effect accounting for pure error, modeled as an independent zero-mean normal distribution with variance *τ^2^*, where and ***I*** is the identity matrix. The elements of the correlation matrix ***H*** are defined by the exponential function as follows:

$$H=exp(-\frac{\delta}{\varphi})$$

where δ represents the distance between two locations, and *φ* is the spatial decay parameter, indicating the distance at which the spatially dependent variance is equal to the sample variance.

For the Bayesian framework, we assigned vague priors to all parameters, including:

$$\beta_{j}\sim N(\beta_{0,j}, M^{-1})$$

$\sigma^{2}= \frac{1}{\lambda_{\sigma}^{2}}$ and $\tau^{2}= \frac{1}{\lambda_{\tau}^{2}}$

$$\lambda_{\sigma}^{2} \sim Gamma(a,b)$$

$$\lambda_{\tau}^{2} \sim Gamma(r,s)$$

$$\varphi\sim U(u,v)$$

Where the regression coefficients **β** follow a multivariate normal distribution with a mean of β_0_​ and a precision matrix of M^−1^. The precisions associated with the variances τ^2^and σ^2^, as well as $\lambda_{\sigma}^{2}$​ and $\lambda_{\tau}^{2}$​follow Gamma distributions, each with shape parameters $a$ and $r$, and scale parameters $b$ and $s$. The spatial decay parameter $\varphi$is uniformly distributed between the minimum $u$ and maximum $v$ distance limits.

The regression model for each process *j* where *j=1* for *An. gambiae*, *j=2* for *An. funestus*, and *j=3* for malaria incidence at spatial location *s* (i.e. longitude and latitude) is expressed as:

$$\log\left( y_{j,s} \right)=Time+ {x_{j,s}}^{T}\beta_{j}+ \omega_{s}+\varepsilon_{s}$$

With Time being an offset term expressed in week of collection (Time = 1, …, 6).

To generate posterior distributions, we used an adaptive Markov Chain Monte Carlo (AMCMC) algorithm (Finley, Banerjee, and E.Gelfand 2015), which runs within a Metropolis-Hastings framework (Sahu 2021) (Sahu, 2022). Joint modeling was achieved by enforcing a shared ω specification across all three models; which was optimized using a metabayes approach (AlShammari, Elgabli, and Bennis 2021), fully described in Kouame et al. 2023. The AMCMC was executed for 1500 iterations after a burn-in of 200 iterations. Convergence was evaluated using Gelman and Rubin's potential scale reduction factor and the Geweke time-series statistics (Du et al. 2022). 95% credible intervals were calculated for each parameter estimate using equal-tailed intervals.
